# AKT signaling is associated with epigenetic reprogramming via the upregulation of TET and its cofactor, alpha-ketoglutarate during iPSC generation

**DOI:** 10.1186/s13287-021-02578-1

**Published:** 2021-09-25

**Authors:** Yoichi Sekita, Yuki Sugiura, Akari Matsumoto, Yuki Kawasaki, Kazuya Akasaka, Ryo Konno, Momoka Shimizu, Toshiaki Ito, Eiji Sugiyama, Terushi Yamazaki, Eriko Kanai, Toshinobu Nakamura, Makoto Suematsu, Fumitoshi Ishino, Yoshio Kodera, Takashi Kohda, Tohru Kimura

**Affiliations:** 1 Laboratory of Stem Cell Biology, Department of Biosciences, Kitasato University School of Science, 1-15-1 Kitasato, Minami-ku, Sagamihara-shi, Kanagawa, 252-0373 Japan; 2 Department of Biochemistry, School of Medicine, Keio University, 35 Shinanomachi, Shinjuku-ku, Tokyo, 160-8582 Japan; 3 Department of Epigenetics, Medical Research Institute, Tokyo Medical and Dental University, 1-5-45 Yushima, Bunkyo-ku, Tokyo, 113-8510 Japan; 4 Department of Physics, Kitasato University School of Science, 1-15-1 Kitasato, Minami-ku, Sagamihara-shi, Kanagawa, 252-0373 Japan; 5 Laboratory for Epigenetic Regulation, Nagahama Institute of Bio-Science and Technology, 1266 Tamura-cho, Nagahama-shi, Shiga, 526-0829 Japan; 6 Center for Disease Proteomics, Kitasato University School of Science, 1-15-1 Kitasato, Minami-ku, Sagamihara-shi, Kanagawa, 252-0373 Japan; 7 Laboratory of Embryology and Genomics, Department of Biotechnology, Faculty of Life and Environmental Sciences, University of Yamanashi, 4-4-37 Takeda, Kofu-shi, Yamanashi, 400-8510 Japan

**Keywords:** AKT signal, TET, αKG, DNA demethylation, Reprogramming, iPS cells

## Abstract

**Background:**

Phosphoinositide-3 kinase (PI3K)/AKT signaling participates in cellular proliferation, survival and tumorigenesis. The activation of AKT signaling promotes the cellular reprogramming including generation of induced pluripotent stem cells (iPSCs) and dedifferentiation of primordial germ cells (PGCs). Previous studies suggested that AKT promotes reprogramming by activating proliferation and glycolysis. Here we report a line of evidence that supports the notion that AKT signaling is involved in TET-mediated DNA demethylation during iPSC induction.

**Methods:**

AKT signaling was activated in mouse embryonic fibroblasts (MEFs) that were transduced with OCT4, SOX2 and KLF4. Multiomics analyses were conducted in this system to examine the effects of AKT activation on cells undergoing reprogramming.

**Results:**

We revealed that cells undergoing reprogramming with artificially activated AKT exhibit enhanced anabolic glucose metabolism and accordingly increased level of cytosolic α-ketoglutarate (αKG), which is an essential cofactor for the enzymatic activity of the 5-methylcytosine (5mC) dioxygenase TET. Additionally, the level of TET is upregulated. Consistent with the upregulation of αKG production and TET, we observed a genome-wide increase in 5-hydroxymethylcytosine (5hmC), which is an intermediate in DNA demethylation. Moreover, the DNA methylation level of ES-cell super-enhancers of pluripotency-related genes is significantly decreased, leading to the upregulation of associated genes. Finally, the transduction of TET and the administration of cell-permeable αKG to somatic cells synergistically enhance cell reprogramming by Yamanaka factors.

**Conclusion:**

These results suggest the possibility that the activation of AKT during somatic cell reprogramming promotes epigenetic reprogramming through the hyperactivation of TET at the transcriptional and catalytic levels.

**Supplementary Information:**

The online version contains supplementary material available at 10.1186/s13287-021-02578-1.

## Background

PI3K/AKT signaling is activated downstream of extracellular stimuli such as growth factors and hormones via receptor tyrosine kinases and G-protein-coupled receptors [1]. Activated AKT phosphorylates serine and threonine residues of multiple target proteins, and cellular survival, proliferation, metabolism and growth are promoted as downstream effects of target phosphorylation. Research from our laboratory and others has shown that AKT activation enhances cellular reprogramming or dedifferentiation in vivo and in vitro [2]. Germ cell-specific *Pten*-deficient mice, which display constitutively activated AKT in germ cells, develop testicular teratomas that emerge from dedifferentiated primordial germ cells (PGCs) at the neonatal stage [3]. We have also shown that AKT activation in PGCs increases the efficiency of their dedifferentiation to pluripotent embryonic germ cells (EGCs) in vitro and that AKT can even replace bFGF, a cytokine required for EGC induction under the conventional protocol [4]. AKT activation also improves the efficiency of iPSC production [5].

In the process of iPSC induction, many functions of cellular physiology (e.g., metabolism, the epigenetic state associated with transcriptional networks, morphology and proliferation rates) dramatically change during the shift from a somatic cell state to a pluripotent stem cell state. Among these changes, metabolic remodeling occurs during the very early stage of reprogramming [6, 7]. Within 2 days following the transduction of Yamanaka factors (OCT4, SOX2 and KLF4; OSK), terminally differentiated cells with low glycolytic activity and an intermediate level of mitochondrial oxidative phosphorylation activity show a shift their metabolic state toward high glycolytic and oxidative phosphorylation activities, known as an OXPHOS burst. Recently, cellular metabolic remodeling has been recognized as an important mechanism controlling the pluripotency state by providing not only energy and cellular building blocks but also rate-limiting substrates for enzymes regulating epigenetic modifications, such as αKG and acetyl-CoA [8–11]. Epigenetic reprogramming occurs during the intermediate stage of iPSC generation. Genome-wide DNA hypomethylation activates pluripotency-associated genes, followed by the reestablishment of DNA methylation to repress cell type-specific genes [12]. TET family dioxygenases catalyze the sequential oxidation of 5mC to 5hmC, 5-formylcytosine (5fC) and 5-carboxylcytosine (5caC), ultimately leading to DNA demethylation. Thus, TETs play an essential role in epigenetic reprogramming during iPSC generation [13–15].

In this study, we observed molecular events in cells undergoing reprogramming with activated AKT in the context of cellular metabolism and DNA methylation and hydroxymethylation. Our results illustrate the association of epigenome reprogramming promoted by PI3K/AKT signaling with metabolic remodeling during iPSC generation.

## Methods

### Mice

C57BL/6 mice for maintaining the Oct4-EGFP transgenic mouse line [16, 17] and KSN/Slc nude mice for the teratoma formation assay (see below) were purchased from Japan SLC. All animal care and experimental procedures were carried out in accordance with the Guidelines for Animal Experiments of Kitasato University and were approved by the Institutional Animal Care and Use Committee of Kitasato University (Approval number: SAS2004). The study was carried out in compliance with the ARRIVE guidelines (https://arriveguidelines.org).

### Mouse embryonic fibroblast derivation

MEFs were isolated from Oct4-EGFP transgenic mice [16, 17]. Multiple E13.5 male embryos were mixed and cultured in DMEM supplemented with high glucose (Nacalai Tesque), penicillin–streptomycin (Nacalai Tesque) and 10% FBS (Equitech-Bio).

### Plasmid construction

To construct pMYs-AKT-Mer, the *Bam*HI-*Xho*I fragment from the pCAGGS-myr-AKT-Mer-IRES-EGFPpA plasmid [18] was cloned into the pMYs retroviral vector, which was linearized, and subjected to IRES-GFP cassette removal by *Bam*HI and *Sal*I digestion (pMYs-IRES-GFP was kindly provided by Dr. Kitamura [3, 19]). To construct the pMYs-Idh1 retroviral vector, *Idh1* was amplified from a cDNA library synthesized using in-house-cultured ES cells (c57bl/6 and DBA/2-mixed background) by RT-PCR (forward primer: 5′-AGTTAATTAAGGATCCACCATGTCCAGAAAAATCCAAGG-3′; reverse primer: 5′-TTATTTTATCGTCGACTTAAAGTTTGGCCTGAGCTA-3′). Then, the RT-PCR amplicon was digested with *Bam*HI and *Sal*I, followed by ligation to the pMYs retroviral vector, linearization and removal of the IRES-GFP cassette by *Bam*HI and *Sal*I digestion. We performed site-directed mutagenesis to produce the IDH1-R132H mutant retroviral vector from pMYs-Idh1 using a mutagenesis primer set (forward primer: 5′-CCATCATCATTGGCC*AC*CATGCATATGGGGAC-3′; reverse primer: 5′-GTCCCCATATGCATG*GT*GGCCAATGATGATGG-3′). pMYs-Flag-TET2CD was kindly provided by Dr. Xu of the Chinese Academy of Sciences.

### Retroviral infection and iPSC derivation

The retroviral transduction of the Yamanaka factors (OSK) along with AKT-Mer, IDH1 or IDH1(R132H) was performed following the original protocol with some modifications [20]. Briefly, 3 × 10^6^ plat-E cells were plated in a 60-mm dish 1 day before transfection with 4.5 µg of the pMXs-Oct4, pMXs-Sox2, pMXs-Klf4, pMYs-AKT-Mer, pMYs-Idh1*,* pMYs-Idh1(R132H) or pMYs-Flag-TET2CD plasmid using X-tremeGENE 9 (Merck), following the manufacturer’s instructions. At 48 h posttransfection, the retroviruses in plat-E medium were collected, mixed and filtered via PVDF filters with a 0.45 µm pore size (Merck, Cat# SLHVR33RS). Next, OE-MEFs that had been plated at 3 × 10^5^ cells per 60-mm dish the day before were infected with retroviruses supplemented with 4 µg/mL polybrene (Sigma-Aldrich, Cat# H9268). At 3 dpi, the transfected cells were replated in a 6-well plate with LIF containing ES medium-DMEM (Nacalai Tesque Cat# 08459-64) supplemented with 10% FCS (Gibco, Cat# 10270, Lot# 42Q3056K), 0.1 mM nonessential amino acids (Nacalai Tesque Cat# 06344-56), 2 mM L-glutamine (Nacalai Tesque Cat# 16948-04), 0.1% 2-mercaptoethanol, 100 U/mL LIF (made in the laboratory) and streptomycin/penicillin (Nacalai Tesque Cat# 09367-34). At the same time, 4OHT (Sigma-Aldrich, Cat# H7904) (final concentration, 1 µM) was added to three wells to activate AKT, and ethanol (vehicle control) was added to another 3 wells for the AKT-nonactivated controls. AOA (Sigma-Aldrich, Cat# C13408) (final concentration, 1 mM) or DM-αKG (Sigma-Aldrich, Cat# 349631) (final concentration, 0.8 mM) was added to the culture medium beginning at either 3 dpi, to examine the levels of αKG and 5hmC (Fig. 4f, g) or 7 dpi, to determine reprogramming efficiency (Fig. 4h, i).

### Teratoma formation assay

Established iPSCs (1 × 10^6^) were subcutaneously injected into the dorsal flank of nude mice (Japan SLC). At 4–5 weeks after transplantation, the tumors were dissected from the mice. The teratomas were fixed with 4% paraformaldehyde in PBS and embedded in OCT compound (Sakura Finetek Japan). Sections (7 µm thick) were stained with hematoxylin and eosin.

### Cell cycle analysis

At 7, 8, 9 and 10 dpi, 1 × 10^6^ cells undergoing reprogramming were harvested in tubes, fixed with 1% PFA for 15 min at room temperature, washed with PBS and resuspended in 100 µL PBS. A total of 900 µL of 70% ethanol chilled at − 30 °C was added, with mixing by tapping, followed by 50 µL RNase (50 µg/µL) and 200 µL propidium iodide (100 µg/mL). Next, the mixture was incubated for 30 min at room temperature. The cells were collected by centrifugation (960 × *g* for 5 min), resuspended in 1% FCS in PBS and passed through a mesh. Fluorescein was measured with a Gallios flow cytometer (Beckman Coulter) and analyzed with Kaluza software (Beckman Coulter).

### Proteomic analysis

Three wells per experimental group were analyzed independently. Cells were harvested at 10 dpi by trypsinization and washed with PBS. The cell pellet was stored at − 80 °C until protein extraction. The methods used for protein extraction and trypsin digestion have been described previously [21]. See Additional file 5 for further methodological details.

### Ion chromatography–tandem mass spectrometry for anionic metabolites

Cells from each experimental group were plated in three wells of 6-well plates and analyzed independently as follows. The culture medium from cells undergoing reprogramming at 10 dpi was replaced with DMEM without glucose (Nacalai Tesque) supplemented with 4.5 g/L D-glucose-^13^C_6_ (Sigma-Aldrich), followed by incubation with 5% CO_2_ for 1 h at 37 °C. Then, the medium was aspirated, and the cells were washed in ice-cold PBS three times with chilling, followed by snap freezing in liquid nitrogen. The plates were stored at − 80 °C until metabolite extraction. The methods used for metabolite extraction and cytosolic fractionation to remove mitochondria from cell lysates have been described previously [22, 23]. See Additional file 5 for further details.

### Microarray analysis

Total RNA was extracted using the miRNeasy Mini Kit (QIAGEN) according to the manufacturer’s instructions. Cyanine-3 (Cy3)-labeled cRNA was prepared from 100 ng RNA using the Low Input Quick Amp Labeling Kit (Agilent) according to the manufacturer's instructions, followed by RNeasy column purification (QIAGEN). Dye incorporation and the cRNA yield were checked with a NanoDrop ND-1000 spectrophotometer. Slides were scanned immediately after washing on an Agilent DNA Microarray Scanner (G2565CA) using the one-color scan setting for 8 × 60 k array slides (scan area 61 × 21.6 mm, scan resolution 3 µm, dye channel set to green, green PMT set to 100% and TIFF file dynamic range of 20 bits). The scanned images were analyzed with Feature Extraction Software 10.7.3.1 (Agilent) using the default parameters to obtain background-subtracted and spatially detrended processed signal intensities.

### Immunoblotting

Proteins were extracted using RIPA buffer (50 mM HEPES pH 7.9, 150 mM NaCl, 1% NP-40, 0.5% sodium deoxycholate, 0.1% SDS), followed by protein concentration measurement using a Pierce BCA Protein Assay Kit (Thermo Fisher Scientific). For SDS-PAGE, 20 µg protein was applied per lane. Proteins were detected with anti-TET2 (diluted to 1:1000) (Cell Signaling Technology) and anti-β-ACTIN (diluted to 1:1000) primary antibodies using ImageQuant LAS 4000 (GE Healthcare).

### RNA sequencing

Total RNA was isolated from cells using the AllPrep DNA/RNA Mini Kit (QIAGEN) according to the manufacturer’s instructions. Standard polyA libraries were prepared using the KAPA Stranded mRNA-Seq Kit for Illumina (Kapa Biosystems) according to the manufacturer’s protocol. Libraries were subjected to 50-bp single-end sequencing on the Illumina HiSeq 1500 platform. The sequence data were trimmed using Trim Galore (https://www.bioinfor matics.babraham.ac.uk/projects/trim_galore/) and mapped against the mouse reference transcriptome using Salmon [24].

### qRT-PCR

Total RNA was isolated from cells using the RNeasy Mini Kit (QIAGEN) according to the manufacturer’s instructions. Reverse transcription was performed by using a ThermoScript RT-PCR System (Invitrogen) and an oligo (dT) primer. Gene expression levels were measured with Stratagene MX3000P (Agilent Technologies) using FastStart Universal SYBR Green Master Mix (Rox) (Roche). See Additional file 5: Table S1 for the primers.

### Bisulfite sequencing

Genomic DNA was isolated from cells using the Cica Geneus Total DNA Prep Kit (Kanto Chemical) according to the manufacturer’s instructions. The isolated DNA was subjected to the bisulfite reaction as described previously with some modifications [25, 26]. The automated shell scripts and the read me text are available in Additional files 2, 3 and 4 [27]. See Additional file 5 for further details.

### Dot blot assay

Total DNA was extracted from cells by RNase and protease digestion, followed by phenol/chloroform purification. The DNA concentration was measured using a Qubit system (Thermo Fisher Scientific) and diluted to 100 ng/µL with distilled and deionized water. To denature the genomic DNA, 0.5 µL of 1 N NaOH was added to 4.5 µL of DNA solution, after which the mixture was heated to 95 °C for 10 min and neutralized by adding 0.75 µL of 5 M ammonium acetate. The genomic DNA (1.5 µL) was blotted onto a nylon membrane (Hybond-N + , GE Healthcare) (i.e., 117 ng genomic DNA per spot). The membrane was incubated at 80 °C for 30 min and UV cross-linked at 70 mJ. The blotting membrane was soaked in blocking reagent (BlockingOne, Nacalai Tesque) for 1 h at room temperature, washed briefly with TBS with 0.1% Tween-20 (TBST), incubated with a polyclonal anti-5hmC antibody (Activemotif, cat # 39069) diluted 1:5000 with Can Get Signal Solution 1 (Toyobo Life Science) at 4 °C overnight, washed with TBST three times for 10 min each, incubated with an HRP-linked anti-rabbit IgG antibody (GE Healthcare, cat # NA934) for 1 h at room temperature and washed with TBST three times for 10 min each. The resulting signals were detected by ECL chemiluminescence (Nacalai Tesque) using ImageQuant LAS 4000 (GE Healthcare). The signal intensity of the dots was quantified using ImageJ software. To plot a standard curve of the dot signal intensity versus the amount of 5hmC, we used a synthesized oligonucleotide consisting of 57 nucleotides including seven 5hmCs (5′-AGAAT[X_1_]GGTTATAGG[X_2_]GGGAGACATAGAAACTGC[X_3_]G[X_4_]GTG[X_5_]GTG[X_6_]GTCCAC[X_7_]GAAAC-3′, [X]; 5hmC). Twofold dilutions of the oligonucleotide were prepared starting from the highest 5hmC concentration of 400 fmol/µL and treated in the same way as the genomic samples described above.

### Statistical analysis

To evaluate reprogramming efficiency, iPSCs from each group were plated in three wells of 6-well plates. Then, the GFP-positive colonies in each well were counted. For dot blotting, transcriptomic, proteomic and metabolomic analyses, qRT-PCR, and bisulfite sequencing, cells from the three wells per experimental group were assessed independently. Statistical analyses of the iPSC generation assay, dot blot analysis, metabolomic analysis, qRT-PCR and methylation analysis data were performed using the unpaired Student’s t-test. Statistical analyses of the RNA sequencing data were performed using the edgeR package [28], and *p* values were calculated after adjusting for the false discovery rate. Data are shown as the means, and error bars represent standard deviations.

### Data and software availability

The accession numbers of the RNA-seq data and the microarray data reported in this paper are GEO: GSE161344 and GEO: GSE161399, respectively. The accession number of the proteomic analysis reported in this paper Japan ProteOme Standard Repository (JPOST; https://repository.jpostdb.org/) is PXD014856. The metabolomic analysis data reported in this paper are available in Additional file 1.

## Results

### Conditional activation of AKT enhances iPSC induction efficiency

**Fig. 1:**
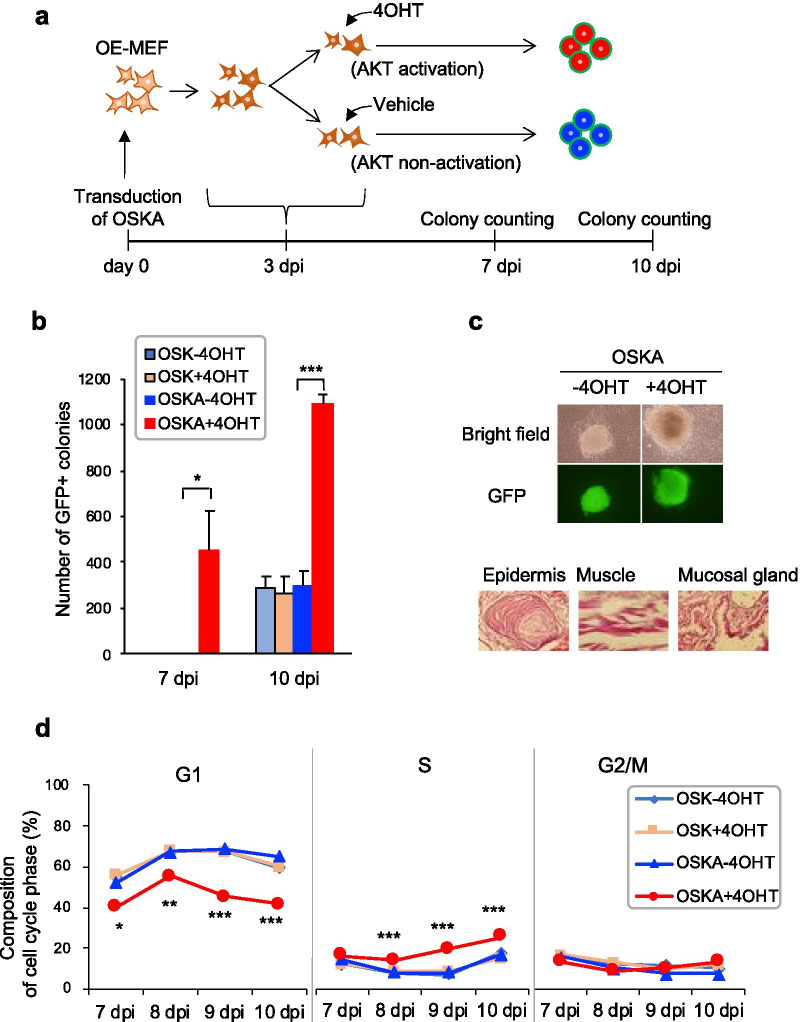
Enhancement of iPSC induction by conditional AKT activation**. a** A schematic representation of conditional AKT activation during iPSC induction. OE-MEFs were transduced with reprogramming factors (OSK) and AKT-Mer (A). Cells were plated in 6-well plates at 3 dpi. Next, 4OHT (the Mer ligand) was added to three wells to activate AKT, and vehicle (ethanol) was added to the remaining three wells as a control. GFP-positive colonies were counted at 7 and 10 dpi. **b** The numbers of GFP-positive colonies at 7 and 10 dpi. Cells without AKT-Mer transduction (OSK − 4OHT and OSK + 4OHT) were observed to record the intrinsic effects of 4OHT on reprogramming (n = 3). **c** Representative images of GFP-positive colonies and histological sections illustrating the three germ layers that differentiated from AKT-activated iPSCs transplanted into nude mice. The epidermis, muscle and mucosal gland correspond to the ectoderm, mesoderm and endoderm, respectively. **d** Cell cycle analysis by DNA content quantitation, as assessed by PI staining and flow cytometry between 7 and 10 dpi (n = 3). *p < 0.05; **p < 0.01; ***p < 0.001 by the unpaired Student’s *t*-test

To investigate the role of activated AKT during iPSC induction, we employed a fusion protein of myristoylated AKT and a modified estrogen receptor (AKT-MER) [4]. In this system, AKT can be conditionally activated by administering the MER ligand 4-hydroxytamoxifen (4OHT). For the induction of cell reprogramming, we used mouse embryonic fibroblasts (MEFs) carrying a transgene encoding enhanced green fluorescent protein gene (*EGFP*) under the control of the *Oct4* promoter and enhancer (OE-MEFs) [16, 17]. The OE-MEFs enabled us to identify cells undergoing reprogramming by observing EGFP fluorescence. We transduced the OE-MEFs with AKT-MER in combination with OSK (OSKA) via retroviral vector infection (Day 0). At 3 days postinfection (dpi), we split the cells into two groups, which were cultured with (OSKA + 4OHT: AKT activation) or without 4OHT (OSKA − 4OHT: AKT nonactivation) (Fig. 1a). We assessed the effect of AKT activation on iPSC induction by counting the number of GFP-positive colonies at 7 and 10 dpi. At 7 dpi, the average number of GFP-positive colonies in the OSKA + 4OHT group was 450 ± 179.5 (i.e., the reprogramming efficiency was 0.15 ± 0.06% when the number of infected cells was 3 × 10^5^), whereas no GFP-positive colonies were observed in the OSKA − 4OHT group (n = 3, *p* = 0.045, Student’s t-test) (Fig. 1b). At 10 dpi, there were 1094 ± 33.3 GFP-positive colonies (reprogramming efficiency: 0.36 ± 0.01%) in the OSKA + 4OHT group compared with 295 ± 65.0 (reprogramming efficiency: 0.10 ± 0.02%) in the OSKA − 4OHT group (n = 3, *p* = 0.00033, Student’s t-test) (Fig. 1b). The effects of 4OHT on the cells that were not transduced with AKT-MER were limited in terms of iPSC induction efficiency and gene expression (Figs. 1b and Additional file 5: Fig. S1). We established iPSC lines from GFP-positive colonies in the OSKA + 4OHT group and confirmed their pluripotent ability to differentiate into the three germ layers using a teratoma formation assay (Fig. 1c). The results indicated that the conditional activation of AKT greatly accelerates the timing and improves the efficiency of somatic cell reprogramming by Yamanaka factors.

We examined cell cycle progression with or without AKT activation from 7 to 10 dpi using fluorescence-activated cell sorting to measure DNA contents following propidium iodide (PI) staining. It has been reported that an accelerated cell cycle rate is important for somatic cell reprogramming [29, 30]. Additionally, one of the major effects of AKT on cell physiology is to promote proliferation by phosphorylating the cell cycle checkpoint proteins p21 and p27 [1]. The proportion of G1-phase cells was significantly lower at all assessed time points, whereas that of S-phase cells was significantly higher among AKT-activated cells (OSKA + 4OHT) than nonactivated cells (OSK with and without 4OHT: OSK − 4OHT and OSK + 4OHT, respectively, and OSKA − 4OHT) at 8 dpi (Fig. 1d). These results indicate that activated AKT promotes the G1-to-S phase transition, thereby accelerating the cell cycle during iPSC induction.

### Expanded glucose anabolism during iPSC induction with activated AKT

**Fig. 2:**
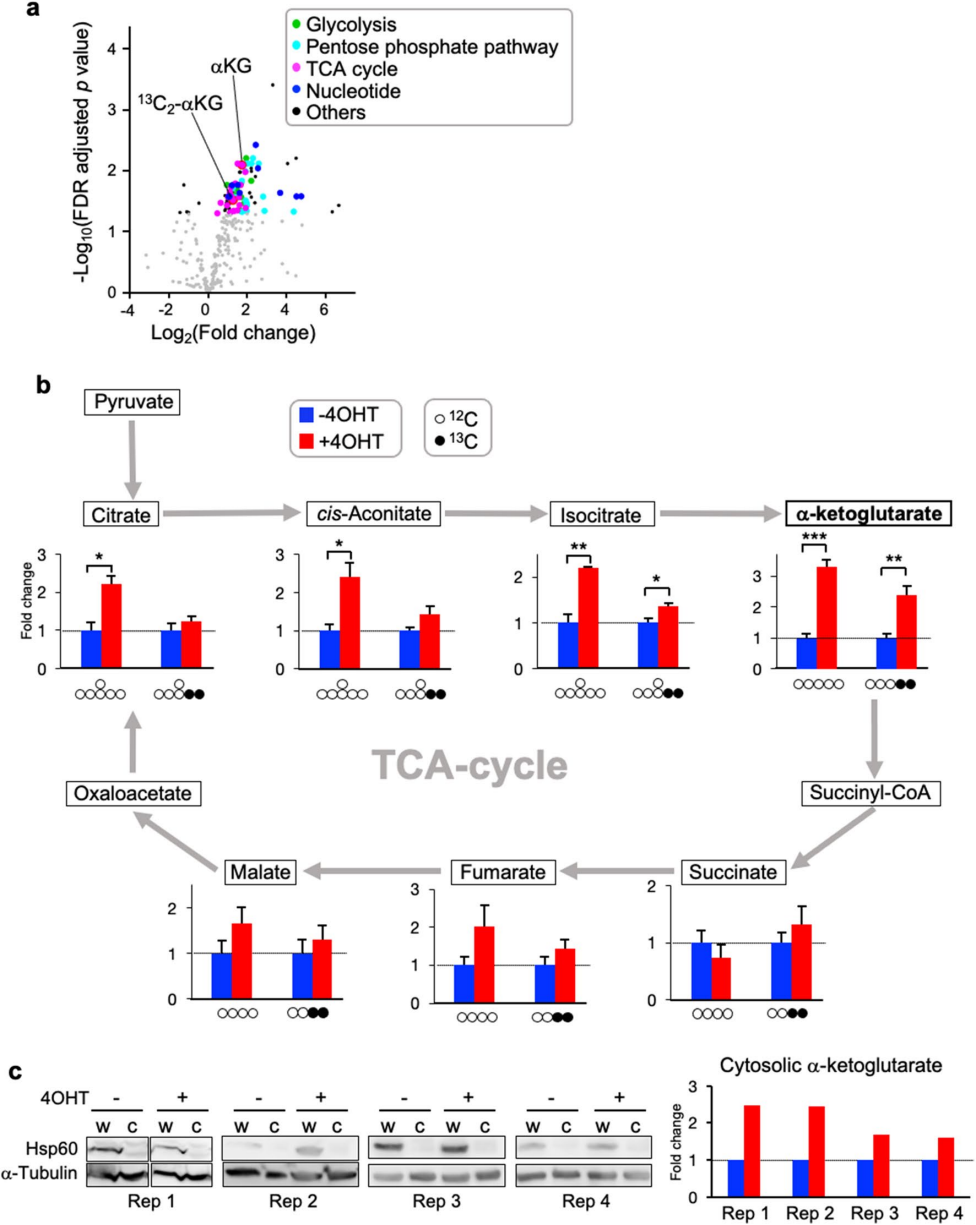
Effects of AKT activation on metabolic pathways during iPSC induction, as assessed by metabolomic analyses. **a** Volcano plot representing all metabolites quantified by ion chromatography–tandem mass spectrometry. For every metabolite, the log_2_ fold change of AKT-activated versus AKT-nonactivated cells was plotted against the − log_10_ false discovery rate adjusted *p* value (n = 3). Colored and black dots depict significantly higher or lower levels of metabolites in AKT-activated cells than in AKT-nonactivated cells, and gray dots represent metabolites showing no significant difference (*p* < 0.05). Each color represents a metabolic process related to processes such as glycolysis, the pentose phosphate pathway, the TCA cycle or nucleotide biosynthesis, as indicated on the upper right. **b** Fold changes in metabolites involved in the TCA cycle, as quantified by ion chromatography–tandem mass spectrometry (n = 3). Metabolites not containing ^13^C were derived from intracellularly accumulated substrates or extracellular nonglucose substrates, whereas ^13^C-containing metabolites were derived from extracellular glucose. The levels of non-^13^C-containing and ^13^C-containing metabolites in AKT-nonactivated cells (− 4OHT) were set to 1. **c** Fold change in cytosolic αKG, as quantified by ion chromatography–tandem mass spectrometry (Rep 1–4; 4 independent experiments). Western blots with antibodies against HSP60 (mitochondrial marker) and α-tubulin (cytosolic marker) indicated the successful exclusion of mitochondria from the cytoplasmic fraction (c) of the whole-cell lysate (w)

As AKT participates in glucose metabolism by regulating GSK and FOXO, we performed a metabolomic pathway tracing analysis by adding ^13^C-labeled glucose to the cell culture medium at 10 dpi. This experiment enabled us to understand how the carbon flows derived from the labeled glucose were modified in each metabolic pathway, where non-^13^C-labeled molecules corresponded to metabolites derived from intracellularly accumulated substrates or extracellular nonglucose substrates. The volcano plot showed an overall increasing trend of the anionic metabolites that we measured (Fig. 2a). Among 313 metabolites, 103 metabolites showed a statistically significant increase, whereas only 5 metabolites showed a statistically significant decrease in AKT-activated cells relative to AKT-nonactivated cells. The flow of glucose carbon into the glycolysis pathway and the pentose phosphate pathway was significantly increased in AKT-activated cells (Additional file 5: Fig. S2a,b). De novo nucleotide synthesis for genome replication, represented by inosine monophosphate (IMP) and deoxynucleotide triphosphates (dGTP, dATP, dCTP and TTP), was notably elevated (Additional file 5: Fig. S2c,d). The carbon flow into the TCA cycle was also significantly increased (Fig. 2b). The levels of intermediates of the TCA cycle, including citrate, cis-aconitate, isocitrate and αKG, significantly altered in AKT-activated cells. Because glucose-derived citrate is exclusively produced in mitochondria, this result suggests a higher level of mitochondrial activity. Importantly, these molecules are involved in fatty acid and amino acid synthesis.

Consistent with the metabolomic analysis, a proteomic analysis at 10 dpi followed by KEGG pathway analysis revealed that proteins involved in glycolysis and the pentose phosphate pathway as well as fatty acid metabolism, amino acid biosynthesis and carbon metabolism were significantly enriched among the proteins upregulated in AKT-activated cells (Additional file 5: Figs S2a,b and S3a,b). Overall, the metabolomic and proteomic analyses showed that iPSC induction with AKT activation shifts the glucose metabolic state toward anabolic processes supplying cellular building blocks (e.g., nucleotides and fatty acids), which could support an accelerated cell cycle rate (Fig. 1d).

### Enhanced epigenetic reprogramming during iPSC induction with activated AKT

**Fig. 3:**
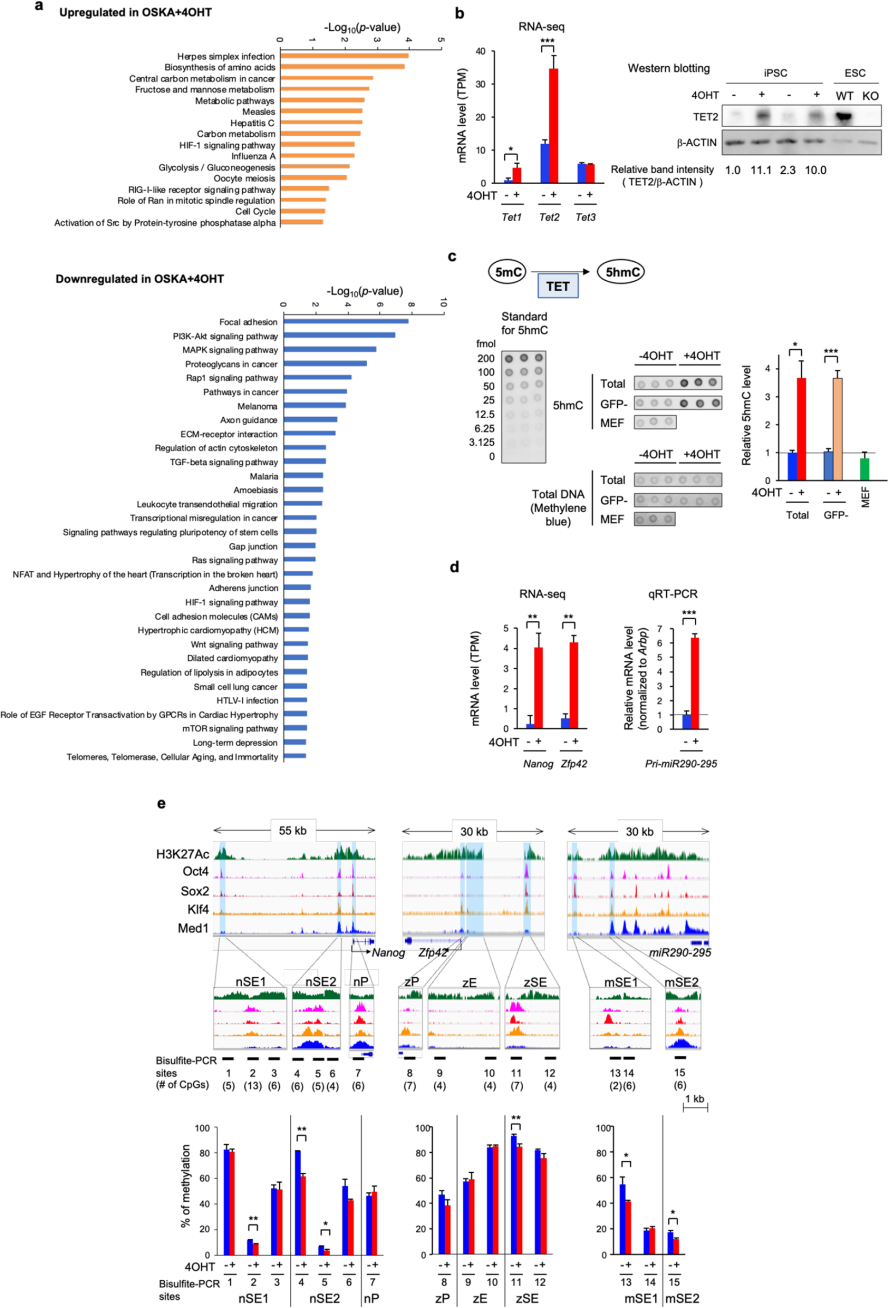
Enhanced DNA methylation reprogramming in AKT-activated cells during reprogramming. **a** RNA-seq followed by the DAVID functional annotation tool used with the KEGG pathway dataset showed that 16 and 32 biological processes were significantly associated with the upregulated and downregulated RNAs, respectively. **b** Transcriptional upregulation of *Tet1* (*p* = 4.9 × 10^−8^) and *Tet2* (*p* = 2.2 × 10^−19^) (n = 3 for each group) by AKT activation at 10 dpi shown by RNA sequencing (left). Upregulation of TET2 at the protein level shown by western blotting (right). **c** Elevated 5hmC levels induced by AKT activation at 10 dpi shown by dot blot analysis. For quantitative analysis, synthesized oligonucleotides containing 5hmC in a known molar amount were plotted. Genomic DNA was extracted from total cells (Total), GFP-negative cells (GFP −) collected by FACS and nontransduced MEFs to measure 5hmC levels (middle and right). The blot was stained with methylene blue after 5hmC detection to assess the total amount of DNA (middle lower panel). **d** Expression levels of pluripotency-related genes measured by RNA sequencing (*Nanog* and *Zfp42*) or qRT-PCR (*pri-miR290-295*) (n = 3). *Pri-miR290-295* expression was normalized to *Arbp* expression, and the level of OSKA − 4OHT was set to 1. **e** DNA methylation levels in cis-regulatory regions around *Nanog* (left column), *Zfp42* (center column) and *pri-miR-290–295* (right column) assayed by bisulfite sequencing adapting PCR amplicon sequencing. The results are presented as the average percentage of methylated CpGs in all sequenced CpGs for each PCR amplicon (n = 3 for each). The top and middle rows are IGV snapshots of the whole regions (top row) and the targeted regions (middle row) depicting publicly available datasets of histone H3K27 acetylation (H3K27Ac) (SRX4178803), the occupancy of the pluripotent stem cell-specific transcription factors Oct4 (SRX950710), Sox2 (SRX1342337) and Klf4 (SRX2441333) and a component of mediator complex Med1 (SRX657147) in ESCs or iPSCs [47]. Blue shading indicates cis-regulatory regions. Bisulfite PCR sites are indicated by bold lines under the IGV snapshots, and the numbers of CpGs involved in each bisulfite PCR amplicon are indicated in parentheses. Abbreviations, SE; super-enhancer, E; enhancer, P; promoter, n; *Nanog*, z; *Zfp42*, m; *pri-miR290-295*

Next, we performed RNA sequencing at 10 dpi. A total of 559 genes were significantly upregulated, and 429 genes were significantly downregulated in the OSKA + 4OHT group relative to the OSKA − 4OHT group (fold change > 2, adjusted *p value* < 0.05, n = 3). KEGG pathway analysis revealed that genes involved in metabolism-related pathways, infection responses and cell cycle regulation were significantly enriched among the genes upregulated in AKT-activated cells (Fig. 3a). On the other hand, genes involved in cell adhesion, motility, the cytoskeleton, cancer and TGF-b signaling were enriched among the downregulated genes (Fig. 3a). The downregulation of TGF-b signaling, which enhances epithelial-to-mesenchymal transition (EMT), suggests that AKT promotes mesenchymal-to-epithelial transition (MET), which is a critical step in reprogramming [31]. Accordingly, *Snai2*, a transcription factor that represses key epithelial regulators, was downregulated in AKT-activated cells (0.67-fold, *p* = 0.04) [31].

Among the differentially expressed RNAs, we focused on the transcriptional upregulation of *Tet1* (5.4-fold, *p* = 0.02) and *Tet2* (2.9-fold, *p* = 9.1 × 10^−5^) in AKT-activated cells (Fig. 3b). This was because in addition to the overexpression of these genes, the elevation of αKG in AKT-activated cells (Fig. 2b,c) led us to speculate that AKT has epigenetic effects, as αKG is an essential cofactor of αKG- and Fe(II)-dependent dioxygenases, including TET enzymes. Consistent with the observed RNA overexpression, an elevated level of the TET2 protein was observed (Fig. 3b).

We investigated the genome-wide level of 5hmC at 10 dpi in bulk cells and GFP-negative cells in the intermediate stage of reprogramming (Fig. 3c). Quantitative dot blot analysis using an anti-5hmC antibody and standards consisting of known amounts of 5hmC-containing oligonucleotides revealed a 3.7-fold increase in the 5hmC level in OSKA + 4OHT bulk cells (n = 3, *p* = 0.015, Student’s t-test; Fig. 3c). We observed a similar upregulation (3.6-fold) of 5hmC in GFP-negative cells collected by fluorescent activated cell sorting (FACS) (n = 3, *p* = 0.0008, Student’s t-test), indicating that the increase in the 5hmC level preceded the activation of the *Oct4-EGFP* reporter gene.

We also investigated the DNA methylation levels of cis-regulatory elements (i.e., promoters and enhancers) of three pluripotency-related genes, *Nanog*, *Zfp42* and *pri-miR290-295*. These genes were significantly upregulated by AKT activation (*Nanog*, 16.4-fold, *p* = 0.006; *Zfp42*, 8.1-fold, *p* = 0.003; *pri-miR290-295*, 6.4-fold, *p* = 2.2 × 10^−5^; Fig. 3d) [32–34]. We collected GFP-negative cells at 10 dpi by FACS and performed bisulfite sequencing by adapting PCR amplicon sequencing using a next-generation sequencer. By comparing the percentage of methylated CpGs in each PCR amplicon, we found a robust decrease in DNA methylation in AKT-activated cells in the ESC super-enhancers of the genes assessed, rather than in their promoter regions (n = 3 for each group; Fig. 3e). Notably, multiple CpGs were observed to be significantly hypomethylated at a single-CpG resolution, whereas no CpGs were significantly hypermethylated in AKT-activated cells (Additional file 5: Fig. S5). Taken together with the TET-mediated genome-wide increase in 5hmC, these findings indicate that pluripotency-related genes are upregulated in AKT-activated cells relative to AKT-nonactivated cells by promoting DNA demethylation at cis-regulatory regions.

### Involvement of increased αKG levels in epigenetic reprogramming during iPSC induction

**Fig. 4:**
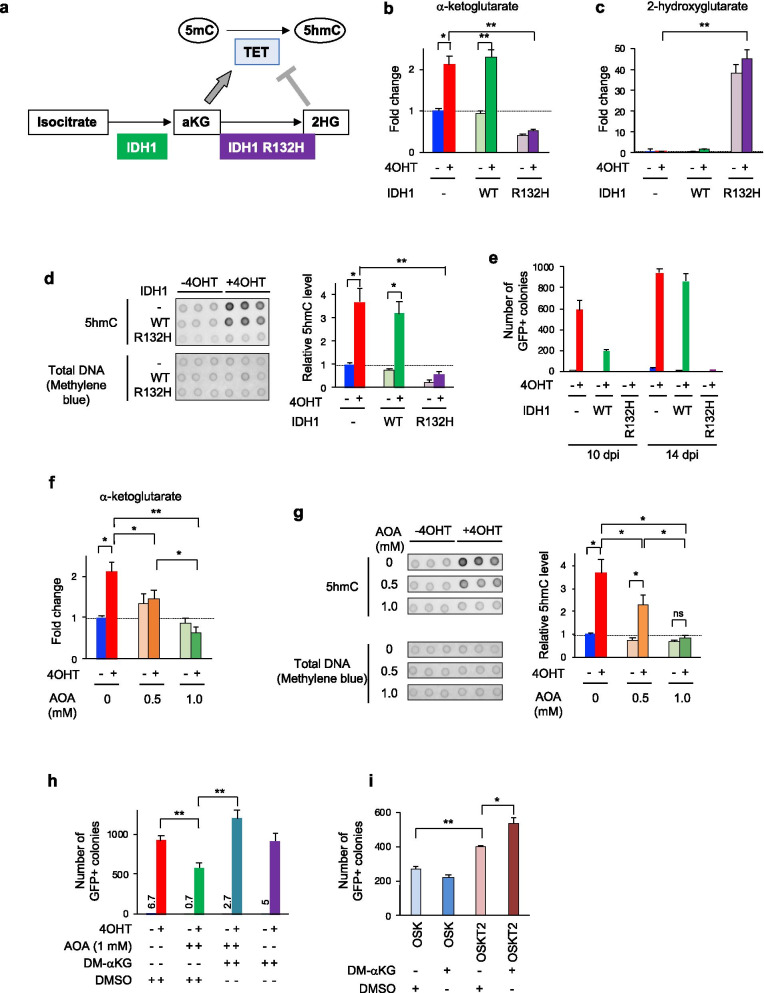
Epigenetic reprogramming promoted by αKG dose-dependent TET activity in AKT-activated cells during reprogramming. **a** A schematic representation of the 5mC-to-5hmC conversion step, including related enzymes (filled shapes) and metabolites (unfilled shapes). αKG is an essential cofactor for TET activity; conversely, 2HG is a TET activity-inhibitory metabolite. **b-e** Requirement of TET-enzymatic activity for an enhanced reprogramming efficiency in AKT-activated cells, as revealed by a decrease in reprogramming resulting from TET inhibition due to 2HG production by IDH1 R132H. Cellular αKG (**b**) and 2HG (**c**) levels in cells undergoing reprogramming induced by OSKA, OSKA + IDH1 and OSKA + IDH1 R132H were quantified by ion chromatography–tandem mass spectrometry at 10 dpi. Genome-wide 5hmC levels were quantified by dot blot analysis (**d**). GFP-positive colonies were counted at 10 and 14 dpi (**e**). **f** and **g** Correlation between αKG levels and genome-wide 5hmC levels at 10 dpi. AOA administration at 3 dpi reduced αKG levels in AKT-activated cells in a dose-dependent manner, as quantified by ion chromatography–tandem mass spectrometry (**f**). Genome-wide 5hmC levels of cells undergoing reprogramming with or without AOA administration at 3 dpi were quantified by dot blot analysis (**g**). **h** Requirement for upregulated αKG production for an enhanced reprogramming efficiency in AKT-activated cells. GFP-positive colonies were counted at 10 dpi. AOA administration at 7 dpi reduced the reprogramming efficiency, but the simultaneous administration of DM-αKG rescued the reprogramming efficiency. **i** Effects of DM-αKG on the reprogramming efficiency with or without TET2CD overexpression. GFP-positive colonies were counted at 14 dpi. The dot blot images shown in Figs. 3 and 4 are from the same membrane as shown in Additional file 5: Fig. S4. Therefore, the ‘total’ in Fig. 3b, ‘IDH1-’ in **d** and ‘AOA-’ in **g** are the same as ‘OSKA’ shown in Additional file 5: Fig. S4

To determine whether the elevated levels of 5hmC in AKT-activated cells were associated with TET activity and pluripotency acquisition, we transduced mutant isocitrate dehydrogenase 1 (IDH1-R132H) together with OSKA to inhibit TET activity. IDH1 functions in the cytosol, and normal IDH1 (IDH1-WT) converts isocitrate into αKG, whereas IDH1-R132H converts αKG into 2-hydroxyglutarate (2HG), which inhibits TET activity [35] (Fig. 4a). We measured cellular αKG and 2HG and genome-wide 5hmC levels at 10 dpi. We also assessed the reprogramming efficiency at 10 and 14 dpi. The level of αKG was not affected by IDH1-WT but was significantly reduced by IDH1-R132H (Fig. 4b). Conversely, the level of 2HG was greatly increased in IDH1-R132H-transduced cells (Fig. 4c). The level of 5hmC was not affected by IDH1-WT but was decreased in IDH1-R132H-transduced cells (Fig. 4d). The reprogramming efficiency assessed by the number of GFP-positive colonies was significantly decreased by IDH1-R132H at both 10 and 14 dpi (Fig. 4e). The number of GFP-positive colonies was decreased by IDH1-WT at 10 dpi but had recovered by 14 dpi, suggesting that IDH1-WT altered the timing, rather than the efficiency, of somatic cell reprogramming (Fig. 4e). These data indicate that elevated levels of 5hmC depend on TET activity and are associated with an increased reprogramming efficiency. In addition, cell reprogramming with activated AKT increases cytosolic αKG during iPSC induction because IDH1-R132H, which functions in the cytosol but not in mitochondria, prevents the increase in the αKG level induced by AKT activation [36] (Fig. 4b). This was confirmed by measuring αKG in the cytosolic fraction without mitochondria (Fig. 2c).

Because TET2 is indispensable for iPSC generation and TET1 or TET2 overexpression during iPSC induction improves the reprogramming efficiency, the elevated expression of *Tet1* and *Tet2* in cells undergoing OSKA + 4OHT-induced reprogramming is potentially involved in the enhanced reprogramming efficiency [13, 37, 38]. Additionally, it is highly possible that the elevated production of αKG is critical for enhanced reprogramming in the OSKA + 4OHT group. To investigate this possibility, we evaluated aminooxyacetic acid (AOA), which is an inhibitor of aminotransferases and decreases cellular αKG levels [10]. AOA administration beginning at 3 dpi prevented the increase in the αKG level in AKT-activated cells (OSKA + 4OHT) in a dose-dependent manner (Fig. 4f). We also found that the increase in 5hmC in the OSKA + 4OHT group was suppressed by AOA administration in a dose-dependent manner (Fig. 4g). Taken together, these data show a strong correlation between αKG levels and genome-wide 5hmC levels. To assess the effect of AOA on the reprogramming efficiency, we administered AOA to cells at 7 dpi because its administration at 3 dpi interfered with cell proliferation. AOA administration significantly decreased the number of GFP-positive colonies at 10 dpi (Fig. 4h). This decrease was rescued by the simultaneous administration of dimethyl-αKG (DM-αKG), a cell membrane-permeable αKG derivative (Fig. 4h). However, DM-αKG administration in AKT-nonactivated cells (OSKA − 4OHT) did not improve the reprogramming efficiency (Fig. 4h), suggesting that an increase in the αKG level in conjunction with the upregulation of TET genes is critical for promoting reprogramming. Therefore, we investigated the effect of DM-αKG in cells undergoing reprogramming with TET2 catalytic domain (TET2CD) overexpression (OSKT2). The OSKT2 group showed a higher reprogramming efficiency than the OSK group at 14 dpi (Fig. 4i). Moreover, DM-αKG administration in the OSKT2 group beginning at 3 dpi significantly improved the reprogramming efficiency (Fig. 4i). Taken together, these results suggest that enhanced 5hmC production and the consequent promotion of somatic cell reprogramming are associated with the synergistic effect of TET overexpression and αKG overproduction in AKT-activated cells.

## Discussion

In this study, we found that glycolysis was enhanced in AKT-activated cells undergoing reprogramming, leading to the accumulation of the downstream TCA cycle metabolite αKG during iPSC induction by OSK. Additionally, while it has been reported that *Tet1* and *Tet2* expression is induced during iPSC induction, we observed higher expression of *Tet1* and *Tet2* in AKT-activated cells than in AKT-nonactivated cells [13, 39]. We speculate that the increases in αKG and TET at least partly account for the genome-wide elevation of 5hmC as well as DNA hypomethylation at ESC super-enhancers, for the following reasons. First, the levels of αKG were correlated with the genome-wide 5hmC levels, suggesting that αKG may play a role in DNA demethylation during somatic cell reprogramming. Second, the inhibition of αKG production abrogated iPSC induction. Furthermore, we showed that simultaneous TET2CD transduction and DM-αKG administration synergistically enhanced the reprogramming efficiency; thus, they partially reproduced the effect of AKT activation on reprogramming.

In addition, these results support the idea that AKT signaling is involved in the enhanced production of TET and αKG. The possible mechanisms of this effect include the following. First, TET overexpression may be induced by introducing Yamanaka factors, as *Tet1* and *Tet2* have super-enhancers with OCT4 binding sites [40], and it has been reported that the AKT-mediated phosphorylation of OCT4, SOX2 and KLF4 upregulates their activity [41, 42]. Second, αKG overproduction involves the AKT-FOXO1 axis [5]. AKT inactivates FOXO1 through direct phosphorylation, which activates glycolysis and mitochondrial function and may result in αKG overproduction [43]. Alternatively, because TET expression was found to be upregulated in cells undergoing reprogramming, these observations might reflect the consequences of an increase in reprogramming mediated by AKT. Further studies are required to reveal the direct and/or indirect linkages among AKT activation, TET expression and αKG production.

Which pathway is responsible for the overproduction of cytosolic αKG is another intriguing question (Fig. 2c). In this study, we used labeled glucose to show that the glucose-derived αKG level was elevated. Glucose-derived αKG accumulates in the cytosol via two pathways: (1) cytosolic citrate transferred from the mitochondria via citrate carrier proteins expressed on the mitochondrial membrane is metabolized to αKG by IDH1, and (2) αKG produced as a TCA cycle intermediate in the mitochondria is transferred to the cytosol via the malate–aspartate shuttle, in which aspartate and αKG are transferred from mitochondria to the cytosol instead of malate and glutamate being transferred from the cytosol to mitochondria. Although it is unknown which of these mechanisms is responsible for the increased level of cytosolic αKG induced in AKT-activated cells, mitochondrial activity is obviously important in both cases. It has been proposed that the balance between glycolysis and mitochondrial activity (e.g., OXPHOS burst) is critical for somatic cell reprogramming [7]. However, the role of mitochondria in cell reprogramming remains unclear [44]. Our findings suggest that mitochondrial activity is important for αKG production because the metabolites of the initial steps of the TCA cycle, especially citrate, were observed to be increased in AKT-activated cells. As the next step, it will be important to elucidate the mechanisms whereby AKT enhances αKG production during reprogramming.

On the other hand, the impact of activated AKT on the reprogramming efficiency is greater than that of the synergistic effect of TET2CD and DM-αKG, indicating that another effect of AKT activation on epigenetic regulation is involved in reprogramming enhancement. It has been proposed that cells with an accelerated cell cycle are dominantly reprogrammed [30]. In this study, we revealed an accelerated G1–S transition. The metabolic shift toward anabolic processes facilitates this phenomenon by providing the cellular building blocks necessary for cell division (i.e., nucleotides for DNA synthesis and phospholipids as a component of the plasma membrane). Moreover, AKT suppresses Chk1, p21 and p27, which are negative regulators of the cell cycle via phosphorylation, and phosphorylates Mdm2 to repress p53 [1]. Through RNA-seq followed by pathway analysis, we also found that MET is promoted in AKT-activated cells, which is associated with the improvement of the reprogramming efficiency by AKT [31]. Several other roles of AKT in cellular reprogramming and pluripotency, including apoptosis inhibition and the respiratory shift mediated by activated AKT in mitochondria, have also been proposed[45, 46]. Altogether, our results showing the role of AKT in epigenetic reprogramming associated with metabolic remodeling add new insight into the wide-ranging roles of AKT signaling in cellular reprogramming.

## Conclusion

In summary, we performed a multiomics analysis to observe the effects of AKT activation on somatic cell reprogramming. These findings may provide novel insights into the dynamic link between AKT signaling and epigenetic regulation mediated by metabolic remodeling in pluripotency acquisition.

## Supplementary Information

**Additional file 1: Data 1 :** Metabolome data for OSKA − 4OHT and OSKA + 4OHT at 10 dpi.

**Additional file 2: ** Auto ma ted shell scr ipt used for quantification of DNA methylation.

**Additional file 3**: Automated shell script used for quantification of DNA methylation for Linux.

**Additional file 4**: Read me for automated shell script used for quantification of DNA m ethylation.

**Additional file 5**: Supplementary Methods, References, Tables and Figures.

## Data Availability

The datasets used and/or analyzed during the current study are available from public databases (see Methods), supplementary information and the corresponding author on reasonable request.

## Abbreviations

PI3K: Phsophoinositide-3 kinase
iPSCs: Induced pluripotent stem cells
PGCs: Primordial germ cells
MEFs: Mouse embryonic fibroblasts
EGFP: Enhanced green fluorescent protein
OE-MEFs: Mouse embryonic fibroblasts carrying *Oct4-EGFP* transgene
dpi: Days postinfection
FACS: Fluorescent activated cell sorting
αKG: α-Ketoglutarate
DM-αKG: Dimethyl-α-ketoglutarate
5mC: 5-Methylcytosine
5hmC: 5-Hydoxymethylcytosine
5fC: 5-Formylcytosine
5caC: 5-Carboxylcytosine
4OHT: 4-Hydroxytamoxyfen
OSK: OCT4, SOX2 and KLF4
AKT-MER: Fusion protein of myristoylated AKT and modified estrogen receptor
OSKA: OCT4, SOX2, KLF4 and AKT-MER
TET2CD: TET2-catalytic domain
OSKT2: OCT4, SOX2, KLF4 and TET2-catalytic domain
IDH1: Isocitrate dehydrogenase 1
2HG: 2-Hydroxyglutarate
AOA: Aminooxyacetic acid

## References

[1] Manning BD, Toker A (2017). AKT/PKB signaling: navigating the network. Cell.

[2] Sekita Y, Nakamura T, Kimura T (2016). Reprogramming of germ cells into pluripotency. World J Stem Cells.

[3] Kimura T, Suzuki A, Fujita Y, Yomogida K, Lomeli H, Asada N (2003). Conditional loss of PTEN leads to testicular teratoma and enhances embryonic germ cell production. Development.

[4] Kimura T, Tomooka M, Yamano N, Murayama K, Matoba S, Umehara H (2008). AKT signaling promotes derivation of embryonic germ cells from primordial germ cells. Development.

[5] Yu Y, Liang D, Tian Q, Chen X, Jiang B, Chou B (2014). Stimulation of somatic cell reprogramming by ERas-Akt-FoxO1 signaling axis. Stem Cells.

[6] Folmes CDL, Nelson TJ, Martinez-Fernandez A, Arrell DK, Lindor JZ, Dzeja PP (2011). Somatic oxidative bioenergetics transitions into pluripotency-dependent glycolysis to facilitate nuclear reprogramming. Cell Metab.

[7] Kida YS, Kawamura T, Wei Z, Sogo T, Jacinto S, Shigeno A (2015). ERRs mediate a metabolic switch required for somatic cell reprogramming to pluripotency. Cell Stem Cell.

[8] Miyazawa H, Aulehla A. Revisiting the role of metabolism during development. Development. 2018;145:dev131110. 10.1242/dev.131110 10.1242/dev.131110 30275240

[9] Tischler J, Gruhn WH, Reid J, Allgeyer E, Buettner F, Marr C, et al. Metabolic regulation of pluripotency and germ cell fate through α‐ketoglutarate. EMBO J. 2019;38:e99518.10.15252/embj.201899518 10.15252/embj.201899518 PMC6315289 30257965

[10] TeSlaa T, Chaikovsky AC, Lipchina I, Escobar SL, Hochedlinger K, Huang J, et al. α-Ketoglutarate accelerates the initial differentiation of primed human pluripotent stem cells. Cell Metab. 2016;24:485–93. 10.1016/j.cmet.2016.07.002 10.1016/j.cmet.2016.07.002 PMC5023506 27476976

[11] Tohyama S, Fujita J, Hishiki T, Matsuura T, Hattori F, Ohno R (2016). Glutamine oxidation is indispensable for survival of human pluripotent stem cells graphical. Cell Metab.

[12] Milagre I, Stubbs TM, King MR, Spindel J, Santos F, Krueger F (2017). Gender differences in global but not targeted demethylation in iPSC reprogramming. Cell Rep.

[13] Doege CA, Inoue K, Yamashita T, Rhee DB, Travis S, Fujita R (2012). Early-stage epigenetic modification during somatic cell reprogramming by Parp1 and Tet2. Nature.

[14] Schwarz BA, Cetinbas M, Clement K, Walsh RM, Cheloufi S, Gu H (2018). Prospective isolation of poised iPSC intermediates reveals principles of cellular reprogramming. Cell Stem Cell.

[15] Sardina JL, Collombet S, Tian TV, Gómez A, Di Stefano B, Berenguer C (2018). Transcription factors drive Tet2-mediated enhancer demethylation to reprogram cell fate. Cell Stem Cell.

[16] Yeom YI, Fuhrmann G, Ovitt CE, Brehm A, Ohbo K, Gross M (1996). Germline regulatory element of Oct-4 specific for the totipotent cycle of embryonal cells. Development.

[17] Yoshimizu T, Sugiyama N, De Felice M, Yeom Y, Ohbo K, Masuko K (1999). Germline-specific expression of the Oct-4/green fluorescent protein (GFP) transgene in mice. Dev Growth Differ.

[18] Watanabe S, Umehara H, Murayama K, Okabe M, Kimura T, Nakano T (2006). Activation of Akt signaling is sufficient to maintain pluripotency in mouse and primate embryonic stem cells. Oncogene.

[19] Murayama K, Kimura T, Tarutani M, Tomooka M, Hayashi R, Okabe M (2007). Akt activation induces epidermal hyperplasia and proliferation of epidermal progenitors. Oncogene.

[20] Takahashi K, Yamanaka S (2006). Induction of pluripotent stem cells from mouse embryonic and adult fibroblast cultures by defined factors. Cell.

[21] Suzuki S, Kodera Y, Saito T, Fujimoto K, Momozono A, Hayashi A (2016). Methionine sulfoxides in serum proteins as potential clinical biomarkers of oxidative stress. Sci Rep.

[22] Clayton DA, Shadel GS (2014). Isolation of mitochondria from tissue culture cells. Cold Spring Harb Protoc.

[23] Miyajima M, Zhang B, Sugiura Y, Sonomura K, Guerrini MM, Tsutsui Y (2017). Metabolic shift induced by systemic activation of T cells in PD-1-deficient mice perturbs brain monoamines and emotional behavior. Nat Immunol.

[24] Patro R, Duggal G, Love MI, Irizarry RA, Kingsford C (2017). Salmon provides fast and bias-aware quantification of transcript expression. Nat Methods.

[25] Sekita Y, Wagatsuma H, Nakamura K, Ono R, Kagami M, Wakisaka N (2008). Role of retrotransposon-derived imprinted gene, Rtl1, in the feto-maternal interface of mouse placenta. Nat Genet.

[26] Kawasaki Y, Kuroda Y, Suetake I, Tajima S, Ishino F, Kohda T. A Novel method for the simultaneous identification of methylcytosine and hydroxymethylcytosine at a single base resolution. Nucl Acids Res. 2017;45:e24. 10.1093/nar/gkw994 PMC5389479 28204635

[27] Kumaki Y, Oda M, Okano M (2008). QUMA: quantification tool for methylation analysis. Nucl Acids Res.

[28] Robinson MD, McCarthy DJ, Smyth GK (2010). edgeR: a Bioconductor package for differential expression analysis of digital gene expression data. Bioinformatics.

[29] Hanna J, Saha K, Pando B, Van Zon J, Lengner CJ, Creyghton MP (2009). Direct cell reprogramming is a stochastic process amenable to acceleration. Nature.

[30] Guo S, Zi X, Schulz VP, Cheng J, Zhong M, Koochaki SHJ (2014). Nonstochastic reprogramming from a privileged somatic cell state. Cell.

[31] Li R, Liang J, Ni S, Zhou T, Qing X, Li H (2010). A mesenchymal-to-Epithelial transition initiates and is required for the nuclear reprogramming of mouse fibroblasts. Cell Stem Cell.

[32] Blinka S, Reimer MH, Pulakanti K, Rao S (2016). Super-enhancers at the nanog locus differentially regulate neighboring pluripotency-associated genes. Cell Rep.

[33] Zhang S, Deng T, Tang W, He B, Furusawa T, Ambs S (2019). Epigenetic regulation of REX1 expression and chromatin binding specificity by HMGNs. Nucl Acids Res.

[34] Suzuki HI, Young RA, Sharp PA (2017). Super-enhancer-mediated RNA processing revealed by integrative MicroRNA network analysis. Cell.

[35] Xu W, Yang H, Liu Y, Yang Y, Wang P, Kim SH (2011). Oncometabolite 2-hydroxyglutarate is a competitive inhibitor of α-ketoglutarate-dependent dioxygenases. Cancer Cell.

[36] Lewis CA, Parker SJ, Fiske BP, McCloskey D, Gui DY, Green CR (2014). Tracing compartmentalized NADPH metabolism in the cytosol and mitochondria of mammalian cells. Mol Cell.

[37] Gao Y, Chen J, Li K, Wu T, Huang B, Liu W (2013). Replacement of Oct4 by Tet1 during iPSC induction reveals an important role of DNA methylation and hydroxymethylation in reprogramming. Cell Stem Cell.

[38] Di Stefano B, Luis Sardina J, van Oevelen C, Collombet S, Kallin EM, Vicent GP (2014). C/EBPα poises B cells for rapid reprogramming into induced pluripotent stem cells. Nature.

[39] Polo JM, Anderssen E, Walsh RM, Schwarz BA, Nefzger CM, Lim SM (2012). A molecular roadmap of reprogramming somatic cells into iPS cells. Cell.

[40] Sohni A, Bartoccetti M, Khoueiry R, Spans L, Vande Velde J, De Troyer L (2015). Dynamic switching of active promoter and enhancer domains regulates Tet1 and Tet2 expression during cell state transitions between pluripotency and differentiation. Mol Cell Biol.

[41] Lin Y, Yang Y, Li W, Chen Q, Li J, Pan X (2012). Reciprocal regulation of Akt and Oct4 promotes the self-renewal and survival of embryonal carcinoma cells. Mol Cell.

[42] Malak PN, Dannenmann B, Hirth A, Rothfuss OC, Schulze-Osthoff K (2015). Novel AKT phosphorylation sites identified in the pluripotency factors OCT4, SOX2 and KLF4. Cell Cycle.

[43] Wilhelm K, Happel K, Eelen G, Schoors S, Oellerich MF, Lim R (2016). FOXO1 couples metabolic activity and growth state in the vascular endothelium. Nature.

[44] Sone M, Morone N, Nakamura T, Tanaka A, Okita K, Woltjen K (2017). Hybrid cellular metabolism coordinated by Zic3 and Esrrb synergistically enhances induction of naive pluripotency. Cell Metab.

[45] Chen YH, Su CC, Deng W, Lock LF, Donovan PJ, Kayala MA (2019). Mitochondrial Akt signaling modulated reprogramming of somatic cells. Sci Rep.

[46] Hossini AM, Quast AS, Plötz M, Grauel K, Exner T, Küchler J (2016). PI3K/AKT signaling pathway is essential for survival of induced pluripotent stem cells. PLoS ONE.

[47] Oki S, Ohta T, Shioi G, Hatanaka H, Ogasawara O, Okuda Y, et al. ChIP‐Atlas: a data‐mining suite powered by full integration of public ChIP‐seq data. EMBO Rep. 2018;19:e462455. 10.15252/embr.201846255 PMC6280645 30413482

