# Supplementary material for: AKT signaling is associated with epigenetic reprogramming via the upregulation of TET and its cofactor, alpha-ketoglutarate during iPSC generation
Source: Stem Cell Res Ther. 2021 Sep 25;12:510. doi: 10.1186/s13287-021-02578-1 (PMC8467031; doi:10.1186/s13287-021-02578-1)
Supplement: Supplementary file 5 — Additional file 5: Supplementary Methods, References, Tables and Figures. [file 13287_2021_2578_MOESM5_ESM.docx]

**Supplementary methods**

**Detailed proteomic analysis.** Proteins were extracted by adding 50 µL of PTS solution (12 mM SDC, 12 mM SLS, 200 mM TEAB) to cell pellets, followed by mixing by tapping. Next, the mixture was sonicated using a Bioruptor sonicator (BM Equipment) at high power, with cycles of 30 s on/30 s off for 30 min in ice-cold water. The mixture was then centrifuged at 19,000 × *g* for 15 min at 4 °C. The supernatant was collected, and the protein concentration was measured using a NanoDrop spectrophotometer. The solution was diluted with PTS solution to 1 µg/µL protein, and 20 µL of the solution was used for further treatment. Proteins were reduced and alkylated as follows: 2 µL TCEP (200 mM, reducing agent) was added, followed by incubation at 50 °C for 30 min; next, 2 µL IAA (375 mM, alkylating agent) was added, followed by incubation at room temperature for 30 min; finally, 2 µL L-cysteine (400 mM, IAA absorbance) was added, followed by incubation at room temperature for 10 min in a dark box. Then, 26 µL of the resulting reduced/alkylated sample was digested by adding 2 µL trypsin (100 ng/µL) and 2 µL Lys-C endopeptidase (100 ng/µL), followed by incubation at 37 °C overnight. The reaction was stopped by placing the tube on ice for 10 min. Surfactants were removed from the solution by adding 30 µL of 5% acetonitrile and 15 µL of 5% trifluoroacetic acid, vortexing the mixture, and centrifuging it at 19,000 × *g* for 15 min at 4 °C. The supernatant was collected, desalted, and concentrated by passing through a STAGE tip treated with methanol and 70% acetonitrile/0.1% trifluoroacetic acid. The sample was placed in the pretreated STAGE tip and centrifuged at 1,000 × *g* for 5 min. Next, the STAGE tip was washed with 0.1% trifluoroacetic acid, and the peptides were eluted with 80 µL 50% acetonitrile/0.1% trifluoroacetic acid. The sample was freeze-dried for 1 h, and the pellet was resuspended in 20 µL 3% acetonitrile/0.1% formic acid by mixing for 30 min. Finally, the mixture was centrifuged at 19,000 × *g* for 15 min at 4 °C. The supernatant was analyzed by LC–MS/MS as follows.

Tryptic digests were injected into a C_18_ 0.075 × 120 mm analytical column (Nano HPLC Capillary Column; Nikkyo Technos) configured for an EASY-nLC 1000 HPLC system (Thermo Fisher Scientific). The flow rate of the mobile phase was 300 nL/min; mobile phase A consisted of 0.1% formic acid (FA), and mobile phase B consisted of 0.1% FA/90% acetonitrile. The mobile phase gradient was programmed as follows: 5–25% B (0–120 min), 25–45% B (120–150 min), 45–95% B (150–153 min), and 95% B (153–160 min). The separated peptides were introduced from the HPLC to a Q-Exactive system (Thermo Fisher Scientific) operating in data-dependent mode to automatically switch between full-scan MS and MS/MS acquisition. Full-scan MS spectra (m/z 350–1,000) were acquired in an Orbitrap instrument with a mass resolution of 70,000 at m/z 200 after the accumulation of ions to a 1 × 10^6^ target value. Fractions representing the 12 most intense full-scan peaks with charge states of 2–4 were selected using an isolation window of 2.4 Da and fragmented in the high-energy collisional dissociation cell at a normalized collision energy of 27%. MS/MS spectra were acquired by an Orbitrap mass analyzer with a mass resolution of 17,500 at m/z 200 after the accumulation of ions to a 2 × 10^5^ target value. The ion selection threshold was 4 × 10^4^ counts, and the maximum allowable ion accumulation times were 120 ms for full MS scans and 300 ms for MS/MS spectra. Typical mass spectrometric conditions were as follows: spray voltage 2 kV, no sheath or auxiliary gas flow and dynamic exclusion time of 20 s.

Database searches were performed using the SEQUEST algorithm incorporated into Proteome Discoverer 1.4.0.288 software (Thermo Fisher Scientific). The detected number of peptide spectrum matches (PSMs) for each protein was normalized to the median, and the average of three independent samples was obtained for each experimental group. Keratins were ignored. To avoid over- and underestimation, we classified proteins into three categories: category 1, the change in the protein level was certain; category 2, the change in the protein level was somewhat certain; and category 3, the change in the protein level was not certain. The criteria for each category are as follows.

**Category 1**:

*Proteins upregulated in AKT-activated cells relative to nonactivated cells*. To avoid overestimation, the ratio of the PSM in AKT-activated cells to that in nonactivated cells was ≥ 1.2, the *p* value (Student’s t-test) was < 0.05, and the difference in the average number of PSMs was ≥ 2. To avoid underestimation, the ratio of the PSM in AKT-activated cells to that in nonactivated cells was > 5, and the *p* value (Student’s t-test) was < 0.2.

*Proteins downregulated in AKT-activated cells relative to nonactivated cells*. To avoid overestimation, the ratio of the PSM in nonactivated cells to that in AKT-activated cells was ≥ 1.2, the *p* value (Student’s t-test) was < 0.05, and the difference in the average number of counted spectra was ≥ 2. To avoid underestimation, the ratio of the PSM in nonactivated cells to that in AKT-activated cells was > 5, and the *p* value (Student’s t-test) was < 0.2.

**Category 2**: To avoid overestimation, the *p* value (Student’s t-test) was ≤ 0.01, and the difference in the average PSM was ≤ 2.

**Category 3**: Not classified into either category 1 or 2.

**Details of ion chromatography–tandem mass spectrometry for anionic metabolites.** The plates were removed from the deep freezer and placed on dry ice to prevent thawing. Next, 250 µL of methanol containing 30 µM L-methionine sulfone and 30 µM MES was added to each well. Cells were scraped from the wells and collected in tubes. Then, 125 µL ultrapure water and 400 µL chloroform were added, and the tube was centrifuged at 10,000 × *g* for 15 min at 4 °C. The aqueous phase was subjected to centrifugal filtration through a 5-kDa cutoff filter (Millipore) to remove proteins. The remaining aqueous solution and chloroform layer were evaporated by heating to quantify proteins for normalization, as described below. The solvent (water) was evaporated using a Speed Vac concentrator at 4 °C, followed by the resuspension of the pellet in 30 µL ultrapure water.

For cytosolic fractionation to remove mitochondria from cell lysates, we followed the standard protocol with some modifications[1]. All steps were performed on ice or at 4 °C. Briefly, cells were scraped from three wells of a 6-well plate with 500 µL RSB hypobuffer after PBS washing and transferred to a 1-mL Dounce homogenizer. After allowing the cells to swell for 5–10 min, they were ruptured using a tight-fitting pestle via 5 cycles of 10 strokes each followed by being left to stand for 3 min. The homogenate (500 µL) was transferred to a 1.5-mL centrifuge tube containing 333 µL of 2.5 × MS homogenization buffer. The homogenizer was rinsed with 100 µL of 1× MS homogenization buffer, and the buffer was added to the homogenate (approximately 900 µL in total). For western blotting of markers to confirm fractionation, 100 µL of the homogenate was retained in a new tube as the whole-cell lysate. Nuclei, unbroken cells, and debris were excluded from the lysate by repeating the following steps three times: homogenate centrifugation at 700 × *g* for 5 min and supernatant collection in a new tube. Finally, to remove mitochondria, the lysate was centrifuged at 8000 × *g* for 15 min. The supernatant was collected in a new tube as the cytosolic fraction. To confirm the success of fractionation, the cytoplasmic fraction and the whole-cell lysate were analyzed by western blotting with antibodies against Hsp60 (mitochondrial marker, diluted to 1:1,000) (Cell Signaling) and α-tubulin (cytosolic marker, diluted to 1;1,000) (Cell Signaling). The cytosolic fraction was snap-frozen in liquid nitrogen and stored at -80 °C until metabolome analysis.

For metabolome analysis focused on the central glucose metabolism pathways (glycolysis, the TCA cycle, and the pentose phosphate pathway), anionic metabolites were measured using an orbitrap-type mass spectrometer (Q-Exactive focus, Thermo Fisher Scientific) connected to a high-performance ion chromatography (IC) system (ICS-5000+, Thermo Fisher Scientific), which enabled us to perform highly selective and sensitive metabolite quantification based on the IC separation and Fourier transfer MS principle [2]. The IC was equipped with an anion electrolytic suppressor (Dionex AERS 500, Thermo Fisher Scientific) to convert the potassium hydroxide gradient into pure water before the sample was introduced to the mass spectrometer. Separation was performed using a Thermo Scientific Dionex IonPac AS11-HC, 4-μm particle-size column. The IC flow rate was 0.25 mL/min, which was supplemented post-column with a 0.18 mL/min makeup flow of MeOH. The potassium hydroxide gradient conditions for IC separation were as follows: 1 mM to 100 mM (0–40 min), 100 mM (40–50 min), and 1 mM (50–60 min), with a column temperature of 30 °C. The Q-Exactive focus mass spectrometer was operated in ESI negative mode for all detections. Full mass scanning (m/z 70−900) was performed at a resolution of 70,000. The automatic gain control target was set at 3 × 10^6^ ions, and the maximum ion injection time was 100 ms. Source ionization parameters were optimized with the spray voltage at 3 kV and other parameters as follows: transfer temperature at 320 °C, S-lens level at 50, heater temperature at 300 °C, sheath gas at 36, and Aux gas at 10. The obtained relative abundances of the metabolites were normalized with the internal standard compound (see ref 38 for detail), and proteins were measured via the BCA assay.

**Details of bisulfite sequencing.** DNA was denatured in 300 mM NaOH at 37 °C for 15 min, treated with bisulfite solution (1.8 M sodium disulfite, 88.3 mM hydroquinone, 212 mM NaOH, 18.5% tetrahydrofurfuryl alcohol) at 50 °C for 4 hr, mixed with binding solution (3.7 M guanidine thiocyanate, 7.9 mM Tris-HCl pH 8.0, 7.9 mM EDTA, 15.8% Triton X-100) and then transferred to a ZymoSpin IC column (Zymo Research). The column was centrifuged at 12,000 × *g* for 1 min and washed with 100 µL wash buffer (20 mM Tris-HCl, pH 8.0, 1 mM EDTA, and 70% ethanol). The desulfonate reaction was performed by adding 200 µL desulfonated buffer (200 mM NaOH and 80% ethanol) to the column and leaving it to stand for 15 min. The column was centrifuged at 12,000 × *g* for 1 min and washed with 200 µL wash buffer twice. Finally, DNA was eluted with 50 µL TE buffer.

Bisulfite-treated DNA was amplified by PCR as described previously with some modifications[3]. PCR was performed with KOD-Multi & Epi- (Toyobo) using specific primers and an Illumina sequence adaptor for 35 cycles. Then, the PCR products were cleaned with NucleoMag NGS Clean-up and Size Select (Takara), and the sequencing adaptor was added to the resulting PCR products via five cycles of PCR using the Nextra XT index kit (Illumina). The 2^nd^ PCR products were cleaned up with NucleoMag NGS Clean-up and Size Select (Takara) and sequenced with an Illumina MiSeq system using the MiSeq Reagent Nano Kit v2 (500 Cycles) (Illumina). The sequence reads were analyzed for methylation quantification by using the Quma methylation analysis tool (http://quma.cdb.riken.jp) with an automated shell script[4]. The sequence depth for each sample was >200 reads. The automated shell scripts and their related read me text are available in Additional files 2, 3, and 4. See Supplementary Table S2 for the primers.

**Supplementary references**

1. Clayton DA, Shadel GS. Isolation of mitochondria from tissue culture cells. Cold Spring Harb Protoc. 2014;2014:1109–11.
2. Miyajima M, Zhang B, Sugiura Y, Sonomura K, Guerrini MM, Tsutsui Y, et al. Metabolic shift induced by systemic activation of T cells in PD-1-deficient mice perturbs brain monoamines and emotional behavior. Nat Immunol. 2017;18:1342–52.
3. Kawasaki Y, Kuroda Y, Suetake I, Tajima S, Ishino F, Kohda T. A Novel method for the simultaneous identification of methylcytosine and hydroxymethylcytosine at a single base resolution. Nucl Acids Res. 2017;45:e24.
4. Kumaki Y, Oda M, Okano M. QUMA: quantification tool for methylation analysis. Nucl Acids Res. 2008;36:170–5.

**Supplementary tables**

**Supplementary Table S1:** The primers used for qRT–PCR.

| Target gene |  | Sequence (5'--3') |
| --- | --- | --- |
| *pri-miR290-295* | Forward | ACCTGGCTCCTAGCCACAAACA |
|  | Reverse | GGGCTATTGTAAAGCCCAAAAGGTA |
| *Arbp* | Forward | CAAAGCTGAAGCAAAGGAAGAG |
|  | Reverse | ATTAAGCAGGCTGACTTGGTTG |

**Supplementary Table S2:** The primers used for bisulfite-PCR

| Target region | Site |  | Sequence (5'--3') |
| --- | --- | --- | --- |
| *Nanog*-SE1 | 1 | Forward | TCGTCGGCAGCGTCAGATGTGTATAAGAGACAG TTAAGAAATAGTGTGGGTATAATGGG |
|  |  | Reverse | GTCTCGTGGGCTCGGAGATGTGTATAAGAGACAG CTCCCTTTCCACATATTAAAAC |
| *Nanog*-SE1 | 2 | Forward | TCGTCGGCAGCGTCAGATGTGTATAAGAGACAG GAGTTGTTATAAGAAGGTTGGT |
|  |  | Reverse | GTCTCGTGGGCTCGGAGATGTGTATAAGAGACAG ACTATAAATAAAAACAATTAAAACCTCCTA |
| *Nanog*-SE1 | 3 | Forward | TCGTCGGCAGCGTCAGATGTGTATAAGAGACAG TTATATAGATGGGTGATTGGG |
|  |  | Reverse | GTCTCGTGGGCTCGGAGATGTGTATAAGAGACAG AACTTTATAACCACTTTAAAACCCC |
| *Nanog*-SE2 | 4 | Forward | TCGTCGGCAGCGTCAGATGTGTATAAGAGACAG GAGGGATTTGGTTTTAGGAAG |
|  |  | Reverse | GTCTCGTGGGCTCGGAGATGTGTATAAGAGACAG AAAAAACTAAAATTAAAAACTTTATACTAC |
| *Nanog*-SE2 | 5 | Forward | TCGTCGGCAGCGTCAGATGTGTATAAGAGACAG AGTTAGGGTATAGAGAAATTTTG |
|  |  | Reverse | GTCTCGTGGGCTCGGAGATGTGTATAAGAGACAG AAATACCTTAAACCTAATTAAAACTAAC |
| *Nanog*-SE2 | 6 | Forward | TCGTCGGCAGCGTCAGATGTGTATAAGAGACAG GAATGGGGAGATAAGAGTTATTATAG |
|  |  | Reverse | GTCTCGTGGGCTCGGAGATGTGTATAAGAGACAG AACTTTAAACTCCCAAAAATCCC |
| *Nanog*-P | 7 | Forward | TCGTCGGCAGCGTCAGATGTGTATAAGAGACAG ATAAATAGAGATTTTGGTAGTAAGG |
|  |  | Reverse | GTCTCGTGGGCTCGGAGATGTGTATAAGAGACAG ACCAAATCAACCTATCTAAAAACC |
| *Zfp42*-P | 8 | Forward | TCGTCGGCAGCGTCAGATGTGTATAAGAGACAG GTAGGTTGGAATGTTTAATGAGAAG |
|  |  | Reverse | GTCTCGTGGGCTCGGAGATGTGTATAAGAGACAG ACTAAAATATAACTAAATCTCAAAACC |
| *Zfp42*-E1 | 9 | Forward | TCGTCGGCAGCGTCAGATGTGTATAAGAGACAG GTAAAATAAAATAAAATAAAATGTTTGG |
|  |  | Reverse | GTCTCGTGGGCTCGGAGATGTGTATAAGAGACAG TTTACTAAAAACTACCAAATTACTAC |
| *Zfp42*-E1 | 10 | Forward | TCGTCGGCAGCGTCAGATGTGTATAAGAGACAG GTTTAAGTATAGGGGTTAGAGGG |
|  |  | Reverse | GTCTCGTGGGCTCGGAGATGTGTATAAGAGACAG CACATTTATAAAATCAAAAATCCCC |
| *Zfp42*-SE2 | 11 | Forward | TCGTCGGCAGCGTCAGATGTGTATAAGAGACAG GGGTGGTAGTTTATAAGATTAGG |
|  |  | Reverse | GTCTCGTGGGCTCGGAGATGTGTATAAGAGACAG TTAAATAACCTAAAAACTCTCAAAAACAC |
| *Zfp42*-SE2 | 12 | Forward | TCGTCGGCAGCGTCAGATGTGTATAAGAGACAG ATTTAGAATTAAGTTAAAAGGTAG |
|  |  | Reverse | GTCTCGTGGGCTCGGAGATGTGTATAAGAGACAG AAATAAAACCAACCAATATCAC |
| *pri-miR290-295* | 13 | Forward | TCGTCGGCAGCGTCAGATGTGTATAAGAGACAG GATAGGATGTAGTTTTAGAAGGG |
| -SE1 |  | Reverse | GTCTCGTGGGCTCGGAGATGTGTATAAGAGACAG AAATTCTAAAATCCAAAACTCCC |
| *pri-miR290-295* | 14 | Forward | TCGTCGGCAGCGTCAGATGTGTATAAGAGACAG GGTAAAAGAGAGGTAAGAGTAAAATTTATG |
| -SE1 |  | Reverse | GTCTCGTGGGCTCGGAGATGTGTATAAGAGACAG TTATCAAAACAATAACCATCTTTAC |
| *pri-miR290-295* | 15 | Forward | TCGTCGGCAGCGTCAGATGTGTATAAGAGACAG TTTTATTGAAAATAATGAGGGG |
| -SE2 |  | Reverse | GTCTCGTGGGCTCGGAGATGTGTATAAGAGACAG CACACAAAAACTTTAAACAAATAAAATTC |

**Supplementary figures**

**Supplementary Figure S1: Effect of 4-Hydroxytamoxifen (4OHT) on Gene Expression**

Gene expression profiles in cells undergoing reprogramming (OSK − 4OHT, OSK + 4OHT, OSKA − 4OHT, OSKA + 4OHT) were analyzed by microarray analysis at 8 dpi and compared. The expression patterns of OSK − 4OHT and OSK + 4OHT were well correlated, showing limited effects of 4OHT administration on gene expression.

**Supplementary Figure S2: Relative levels of metabolites related to glycolysis, the pentose phosphate pathway, and nucleotide biosynthesis as quantified by ion chromatography–tandem mass spectrometry (n=3).**

(a and b) Fold changes in metabolites involved in glycolysis (a) and the pentose-phosphate pathway (b). For GAPDH and DHAP or 3PG and 2PG in glycolysis, the sum of each pair of molecules is shown, as they have the same molecular weight and are not distinguishable using this method. The enzymes responsible for each reaction are shown alongside the arrows; the colored backgrounds of the enzymes correspond to the heat map shown on the right.

(c) A schematic representation of the nucleotide biosynthesis pathway.

(d) The levels of metabolites related to nucleotides.

Metabolites in AKT-nonactivated cells (− 4OHT, blue bars) were set to 1 (n=3). * p<0.05; ** p<0.01; *** p<0.001 by the unpaired Student’s t-test.

**Supplementary Figure S3: Differentially expressed proteins between the OSKA − 4OHT and OSKA + 4OHT groups at 10 dpi revealed by LC–MS/MS proteomic analysis.**

The spectral counting method, performed using biological triplicate samples, detected approximately 3900 proteins. Among these proteins, 234 were upregulated and 191 were downregulated in the OSKA + 4OHT group relative to the OSKA − 4OHT group (fold change > 1.4, n=3, see Methods for the details on the assessment of significance).

(a) The DAVID functional annotation tool applied with the KEGG pathway dataset showed that 20 and 16 biological processes were significantly associated with the upregulated and downregulated proteins, respectively. Among the 20 upregulated pathways, 11 were related to metabolic processes (dots to the right of the bars).

(b) Heat maps showing expression differences in proteins related to glycolysis, the pentose phosphate pathway, fatty acid metabolism, amino acid biosynthesis, and carbon metabolism between OSKA − 4OHT and OSKA + 4OHT cells.

**Supplementary Figure S4: Whole-membrane images from the dot blot analysis of 5hmC shown in Figures 3 and 4**

The membrane was stained with methylene blue (lower image) to assess the DNA levels after 5hmC immunoblotting (upper image).

**Supplementary Figure S5: DNA methylation profiles in cis-regulatory regions of pluripotency-related genes (*Nanog*, *Zfp42* and *pri-miR290-295*) at a single-CpG resolution assessed by bisulfite PCR followed by deep sequencing.**

The results are presented as the average percentage of methylation ± S.D. for each CpG site. SE; super-enhancer, E; enhancer, P; promoter. Asterisks indicate statistically significant differences between OSKA − 4OHT and OSKA + 4OHT. *; p<0.05, **; p<0.01, ***; p<0.001.

**Supplementary Figure S6: Whole-membrane images from the western blot analysis of TET2 (the upper image) and β-ACTIN (the lower image) shown in Fig. 3a.**
